# Recovery of Otoacoustic Emission Function in Luetic Endolymphatic Hydrops: A Possible Measure of Improvement in Cochlear Function

**DOI:** 10.1155/2009/942096

**Published:** 2009-06-03

**Authors:** Robert H. Chun, Jayant M. Pinto, Rebecca Blankenhorn, Vijay S. Dayal

**Affiliations:** ^1^Department of Otolaryngology and Communication Sciences, Medical College of Wisconsin, 9000 West Wisconsin Avenue, P.O. Box 1997, Milwaukee, WI 53201, USA; ^2^Department of Surgery, Section of Otolaryngology—Head and Neck Surgery, University of Chicago, Chicago, IL 60637, USA

## Abstract

Syphilis is a preventable and curable multi-organ disease caused by *Treponema pallidum* that may also affect the inner ear. First reported in 1887 by Adam Politzer, luetic endolymphatic hydrops (LEH) is a treatable complication of syphilis which causes a potentially reversible sensorineural hearing loss. Symptoms of LEH include fluctuating hearing loss (often low frequency), tinnitus, and vertigo. Though audiometric parameters have been examined in patients with otosyphilis, few studies have examined the use of otoacoustic emissions (OAEs) as a tool to measure improvement in cochlear function. Here we report an improvement in hearing loss, speech discrimination, and OAEs following treatment of LEH.

## 1. Introduction

Since 2000, the incidence of primary and secondary syphilis in the U.S. has been increasing. During 2005-2006, the number of cases reported to CDC increased 12.4% for early latent syphilis (from 8176 to 9186), 9.9% for late and late latent syphilis (from 16 049 to 17 644), and 11.0% for the total number of cases of syphilis (P&S, early latent, late, late latent, and congenital syphilis) (from 33 288 to 36 935) [1]. Among males, the rate of primary and secondary syphilis has risen 70% over the past five years. Among females, the rate of primary and secondary syphilis increased 11.1% from 2005-2006. Thus, with an increasing national rate, syphilis and its subsequent complications represent an important consideration in the evaluation of neuro-otologic disease.

Unlike primary idiopathic hydrops, treatment of LEH with steroids and antibiotics often reverses the symptoms. In a study by Lindstrom and Cleich, treatment of otosyphilis with penicillin and steroids caused an improvement in hearing (speech reception threshold and/or speech discrimination in 25%), decrease in tinnitus (71%), and improvement in disequilibrium in 66% of their patients [2]. Amenta and Dayal found that the most obvious improvement in cases of treated LEH is the speech discrimination score [3]. However, improvement in cochlear function has not been described in the treatment of LEH.

**Case Presentation**

**Figure 1:**
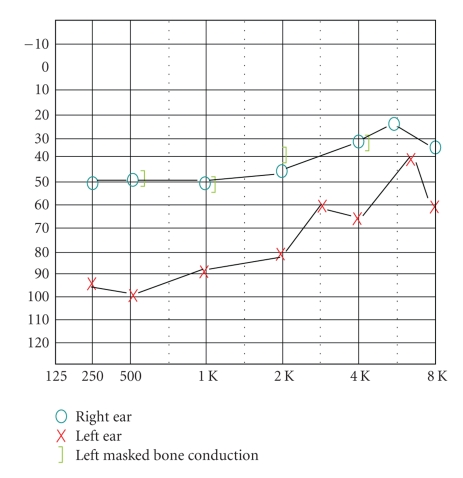
Audiogram prior to treatment.

A 42-year-old female presented with complaints of hearing loss in her left ear for 2-3 months with occasional bilateral tinnitus. Prior to treatment, standard behavioral audiometry (250–8000 Hz) was obtained (Figure 1), as well as distortion product otoacoustic emissions (DPOAEs). DPOAEs were tested using Bio-logic Systems Corp. Scout Otoacoustic Emissions Testing System, software version 3.45.00, diagnostic protocols. Pretreatment results for the patient's right ear indicated moderate to mild sensorineural hearing loss, and the left ear revealed profound to moderate mixed hearing loss (left bone conduction similar to right bond responses). Speech discrimination scores were good in the right ear, 84% at 50 dBHL; poor in the left ear, 60% at 85 dBHL. DPOAE testing revealed distortion product minus noise floor was >5 dB for only 15% of measured points (750–8000 Hz) in the right ear and 32% in the left ear.

**Figure 2:**
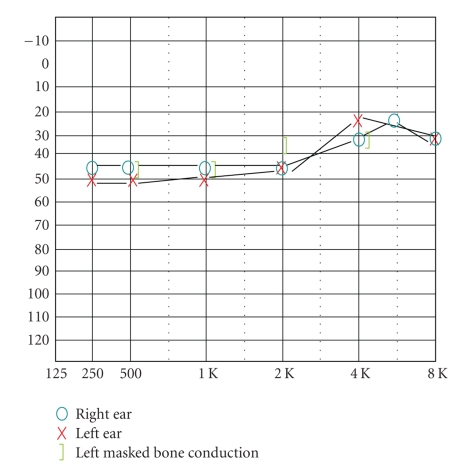
Audiogram following treatment.

Laboratory testing included collagen profile test, RPR, and fluorescent treponemal antibody absorption (FTA-ABS) test. Results revealed the patient's RPR and fluorescent treponemal antibody absorption test to be positive. Furthermore, a history of syphilis was confirmed. Following a course of 2.4 million units of Benzathine Penicillin G and Prednisone for three weeks, the patient reported improvement in hearing. Repeat audiologic evaluation obtained one month after diagnosis revealed improved hearing (Figure 2), and interestingly, the patient's DPOAEs recovered following treatment. Posttreatment results revealed improved left ear responses (mild, flat sensorineural hearing loss with moderate conductive component at 500–1000 Hz). Speech discrimination improved to 92% in the right ear and 96% in the left ear (both presented at 75 dBHL). Posttreatment DPOAEs were measured (2000–8000 Hz) and revealed right ear distortion product minus noise floor was >5 dB for 93% of measured points (range 5.1–15.6 dB) and left ear 86% (range 6.3–15.5 dB), reflecting a recovery of cochlear function. The patient continues to show recovery of both hearing and OAEs nearly one year from treatment.

## 2. Discussion

Syphilis is a multisystem disease that can affect the inner ear. Patients with LEH may present with tinnitus, fluctuating hearing loss with poor speech discrimination, episodic vertigo, and nystagmus [3]. LEH manifests during the late stages of congenital or acquired syphilis. It is known that syphilis within the temporal bone causes a round cell infiltration with endarteritis, resulting in tissue destruction involving any or all of the periosteum, periosteal bone, enchondral bone, and endosteum. With penetration of the endosteum, there is a proliferative fibrosis with obliteration of the perilymphatic space, cochlear hydrops and degeneration of the neuroepithelium of both the cochlear and vestibular end organs [4].

 OAEs are believed to be a measurement of outer hair cell function and to be a by-product of the cochlear amplifier, a metabolic mechanism that enhances cochlear partition vibration during transmission of sound through the hearing system [5, 6]. Recovered OAEs in this patient strongly suggest that OAEs may serve as a tool to measure cochlear function and serve as a treatment outcome for LEH.

In this patient, there was both an improvement in hearing loss as well as recovery of OAE following treatment. This reversal of OAE loss suggests that injury to outer hair cell function and the cochlear amplifier are reversible entities in this disease. We suggest that OAE testing can be used to monitor response to treatment of LEH. Further studies may be indicated to assess the sensitivity and specificity of OAE testing as a therapeutic measurement.
